# Lithiumbehandlung und Senkung des Suizidrisikos: Evidenz und klinische Bedeutung

**DOI:** 10.1007/s00115-025-01880-0

**Published:** 2025-08-15

**Authors:** Michael Bauer

**Affiliations:** Klinik und Poliklinik für Psychiatrie und Psychotherapie, Universitätsklinikum Carl Gustav Carus, Medizinische Fakultät der Technischen Universität Dresden, Fetscherstraße 74, 01307 Dresden, Deutschland

## Epidemiologie von Suiziden

Suizide sind ein vielschichtiges globales Gesundheitsproblem, das jährlich für mehr als 700.000 Todesfälle verantwortlich ist [1]. Im Vergleich zur Allgemeinbevölkerung weisen Patienten mit affektiven Störungen eine erhöhte standardisierte Sterblichkeitsrate (SMR) für alle Todesursachen auf [2]. Psychologische Autopsiestudien in Ländern mit hohem Einkommen haben ergeben, dass mindestens 90 % der Menschen, die durch Suizid sterben, zum Zeitpunkt ihres Todes an einer psychischen Störung litten [3]. Menschen mit psychischen Erkrankungen, insbesondere mit depressiven und bipolaren Störungen, haben ein deutlich erhöhtes Suizidrisiko [4].

## Pharmakologische Beeinflussung von Suizidalität und Suizidrisiko

Die klinische Bewertung des Suizidrisikos sowie die Umsetzung von Strategien zur Suizidprävention sind entscheidende Faktoren bei der Behandlung affektiver Erkrankungen. Leider sind die verfügbaren Therapieoptionen meist empirischer Natur und es fehlen strenge wissenschaftliche Belege. Dies gilt für psychologische, pharmakologische und andere biologische Behandlungsmöglichkeiten gleichermaßen [5]. Ärzte verschreiben in der Regel Antidepressiva, Benzodiazepine und Antipsychotika zur Behandlung akuter Suizidalität. Es gibt jedoch keine Belege dafür, dass eine dieser Medikamentengruppen eine „akut antisuizidale“ oder „suizidpräventive“ Wirkung aufweist. Dies gilt auch für Antidepressiva, da deren Einnahme zumindest vorübergehend zu Beginn der Behandlung bei jungen Menschen unter 25 Jahren zu einer Zunahme der Suizidalität führen kann (was entsprechende Warnhinweise der Food and Drug Administration (FDA) für diese Medikamentengruppe nach sich zog [6]).

Nur für zwei Substanzen, Lithium und Clozapin, gibt es Forschungsergebnisse, die darauf hindeuten, dass die Suizidrate bei einer Langzeitbehandlung reduziert werden kann. Für Clozapin wurden solche Wirkungen allerdings nur bei Schizophrenie untersucht [7]. Lithium ist das Medikament, das in den meisten Studien auf seine suizidpräventive Wirkung untersucht wurde. Seit den frühen 1970er-Jahren bestätigen zahlreiche Berichte und Studien verschiedener Forschungsgruppen aus unterschiedlichen Ländern die Erkenntnis, dass eine langfristige Lithiumtherapie die Suizidrate senken kann (Übersichten: [8, 9]). Für andere stimmungsstabilisierende Medikamente („mood stabilizer“) konnten solche Effekte bislang nicht belegt werden [10].

## Aktueller Stellenwert von Lithium bei affektiven Störungen

Lithium ist das älteste in der Psychopharmakologie immer noch umfänglich eingesetzte Medikament, dessen Geschichte als Wirkstoff zur Behandlung affektiver Erkrankungen bis ins 19. Jahrhundert zurückreicht. Allerdings erfolgte die klinische Anwendung erst ab 1949 auf wissenschaftlicher Basis [11]. In den vergangenen 75 Jahren der Lithiumforschung wurden verschiedene klinische Wirkungen sowie eine Vielzahl neurowissenschaftlicher Erkenntnisse gewonnen. Welche Befunde für die therapeutischen Effekte verantwortlich sind, ist jedoch bis heute unvollständig geklärt. Nach der frühen Entdeckung der antimanischen Wirkungen waren es insbesondere die Entdeckung der Rezidivprophylaxe bei bipolaren Störungen (Metaanalyse: [12]) und die Verstärkung der Wirkung von Antidepressiva (sog. Lithiumaugmentation) bei depressiven Episoden, die Lithium zum Durchbruch verholfen haben. Deshalb wird Lithium international zu Recht als „Goldstandard“ bei der Behandlung bipolarer Störungen bezeichnet, auch wenn es bei langfristiger Anwendung in seltenen Fällen zu einer chronischen Nierenerkrankung kommen kann [13]. In früheren Studien der 1970er-Jahre hat sich zudem gezeigt, dass Lithium auch bei der Prophylaxe rezidivierender unipolarer Depressionen wirksam ist [14].

Im Gegensatz zur aktuellen Entwicklung, in der Lithium in vielen Bereichen des menschlichen Lebens zunehmend genutzt wird, scheint die Verwendung bei der Behandlung bipolarer Störungen zu stagnieren und ist zumindest gemessen an den Verschreibungsdaten rückläufig [15, 16]. Das wird diejenigen, die Lithium als ein „veraltetes Medikament“ betrachten, nicht überraschen. Es steht jedoch außer Frage, dass seine klinische Wirksamkeit im Widerspruch zu den zunehmenden Forschungsergebnissen, umfangreichen klinischen Erfahrungen und den entsprechenden Praxisleitlinien steht [17]. Tatsächlich wird Lithium in allen bedeutenden internationalen Leitlinien zur Behandlung bipolarer Störungen als „First-line“-Medikament bewertet, insbesondere für die Indikation rezidivprophylaktische Langzeittherapie. Hervorzuheben ist, dass die deutschen S3-Leitlinien zur Therapie bipolarer Störungen Lithium als *das einzige Medikament der ersten Wahl* („Lithium first: Not merely first line“) in der Rezidivprophylaxe bewerten [18].

## Lithium und Suizidprotektion: die Anfänge in den 1970er-Jahren

Nachdem erste große longitudinale Studien in den 1960er-Jahren die rezidivprophylaktischen Wirkungen von Lithium beschrieben hatten, waren es die späten 1970er- und 1980er-Jahre, in denen zunächst anekdotische Beschreibungen und naturalistische Studien publiziert wurden, die erstmals auf eine suizidverhindernde Wirkung von Lithium hinwiesen. Diese Beobachtungen wurden von mehreren Forschergruppen in Europa und Kanada aufgegriffen und in systematischeren Untersuchungen weiterverfolgt. Im Jahr 1992 zeigten Forscher der International Group for the Study of Lithium Treated Patients (IGSLi) in einer Studie mit 827 lithiumbehandelten Patienten mit affektiven Störungen, dass deren Mortalität sich nicht von der einer vergleichbaren gesunden Population unterschied [19]. Dabei ist zu berücksichtigen, dass bipolare Störungen mit einer erhöhten Mortalität verbunden sind, wobei die standardisierte Mortalitätsrate mindestens doppelt so hoch ist wie in der Allgemeinbevölkerung [20]. Systematische Beobachtungen von Patientenkohorten, die mit Lithium behandelt und longitudinal verfolgt wurden, ergaben eine verringerte Gesamtmortalität und ein verringertes Risiko für Todesfälle aufgrund von Suizid und kardiovaskulären Ursachen im Besonderen [21].

Felber und Kyber [22] aus Dresden beobachteten eine 10-fache Verringerung der Suizidversuche und eine 3‑fache Verringerung der Suizide bei Patienten unter Lithiumbehandlung im Vergleich zu einer unbehandelten Phase. Eine schwedische Studie mit 362 Patienten mit affektiven Störungen ergab, dass das relative Suizidrisiko bei Patienten ohne Lithiumbehandlung 4,8-mal höher war als bei Patienten unter Lithiumbehandlung [23]. Ahrens und Müller-Oerlinghausen [24] stellten nicht nur bei Patienten, die sehr gut auf Lithium ansprachen (sog. exzellente Lithiumresponder), sondern auch bei Patienten mit mäßigem bis schlechtem Ansprechen auf Lithium eine Verringerung der Suizidversuche fest. Dies ist ein wichtiger Hinweis darauf, dass Lithium unabhängig von seinen stimmungsstabilisierenden und rezidivprophylaktischen Eigenschaften eine suizidverhindernde Wirkung haben könnte und die suizidpräventive Wirkung von Lithium auf einem anderen Mechanismus beruht.

## Epidemiologisch fundierte Befunde

In der Folgezeit lieferten zahlreiche epidemiologische Studien aus unterschiedlichen Ländern weitere überzeugende Belege für die suizidhemmende Wirkung von Lithium. Aus Platzgründen kann im Folgenden lediglich eine Auswahl dargestellt werden.

### USA

Goodwin et al. [25] publizierten eine US-amerikanische Stichprobe von 20.623 krankenversicherten Patienten mit bipolaren Störungen. Die Gruppe, die Lithium erhalten hatte, wies ein um den Faktor 1,5 bis 3 geringeres Risiko für Suizid oder Suizidversuche auf als die Gruppe, die Valproat erhielt.

Collins und McFarland [26] untersuchten 12.662 Medicaid-Patienten in den USA und zeigten, dass unter den mit Lithium behandelten bipolaren Patienten die niedrigste Zahl an Suizidversuchen zu verzeichnen war.

### Schweiz

Die Autoren der longitudinalen Kohortenstudie aus Zürich, in der 406 Patienten mit affektiven Störungen über einen Zeitraum von 40 Jahren beobachtet wurden, berichteten von einer niedrigeren Sterblichkeitsrate bei mit Lithium behandelten Patienten; die Sterblichkeitsrate unter den mit Lithium behandelten Patienten unterschied sich nicht von der der Allgemeinbevölkerung [27].

### Skandinavien

Ein dänisches nationales Register mit 13.186 Patienten, in dem diejenigen, die eine oder mehrere Verschreibungen für Lithium erhalten hatten, mit der Allgemeinbevölkerung verglichen wurden, der nie Lithium verschrieben worden war, ergab, dass Patienten, die mehr als einmal Lithium bezogen, eine niedrigere Suizidrate aufwiesen als diejenigen, die kein Lithium bezogen [28].

Eine weitere Analyse des dänischen Gesundheitsregisters verglich psychotrope Medikamente zur Behandlung bipolarer Störungen und bemerkte, dass Lithium mit einer geringeren Rate an Suiziden, Selbstverletzungen und Wiedereinweisungen in psychiatrische Kliniken verbunden war; außerdem war Lithium bei der Suizidprävention besser wirksam als keine Behandlung [29].

In einer Beobachtungskohortenstudie mit Verknüpfung landesweiter Register aller Personen mit einer Diagnose einer bipolaren Störung in psychiatrischen Krankenhäusern, denen in Dänemark im Zeitraum von 1995 bis 2006 Valproat oder Lithium verschrieben wurde, wurden 4268 Teilnehmer eingeschlossen, von denen 719 nach der Diagnose einer bipolaren Störung Valproat und 3549 Lithium erhielten. Die Gesamtquote der Einweisungen war bei Patienten, die mit Valproat behandelt wurden, nach etwa einem Jahr höher als bei Patienten, die Lithium erhielten, wobei dieser Anstieg bei Patienten mit einer Indexdepression am ausgeprägtesten war [30].

Durch die Verknüpfung mehrerer schwedischer nationaler Register wurden 51.535 Personen mit bipolarer Störung von 2005 bis 2013 hinsichtlich ihrer Behandlung mit Lithium und Valproat beobachtet [31]. Während der Nachbeobachtungszeit traten 10.648 suizidbezogene Ereignisse auf. Die Inzidenzrate sank während der Lithiumbehandlung signifikant um 14 % (Hazard Ratio 0,86), nicht jedoch während der Valproatbehandlung (Hazard Ratio 1,02). Der Unterschied in den Hazard Ratios für suizidbezogene Ereignisse zwischen Lithium und Valproat war statistisch signifikant [31].

### Großbritannien

Eine longitudinale Kohortenstudie an einer national repräsentativen Stichprobe unter Verwendung elektronischer Gesundheitsdaten, die ab 1995 bis 2013 erhoben wurden, umfasste alle Patienten, bei denen eine bipolare Störung diagnostiziert wurde und denen Lithium, Valproat, Olanzapin oder Quetiapin als Stimmungsstabilisator zur Rezidivprophylaxe verschrieben wurde. Im Vergleich der Raten von Selbstverletzung, unbeabsichtigten Verletzungen und Suizid bei Patienten mit bipolarer Störung zeigte Lithium eine deutliche Überlegenheit gegenüber den anderen Substanzen [32].

## Systematische Übersichten

Eine der ersten systematischen Übersichtsarbeiten zeigte in einer gepoolten Stichprobe von mehr als 17.000 Personen, dass das Suizidrisiko bei mit Lithium behandelten Patienten um das 8,6-Fache geringer war als bei Patienten, die kein Lithium einnahmen [33]. Eine Metaanalyse von 31 klinischen Publikationen derselben Forschergruppe, darunter 5 randomisierte kontrollierte Studien, berichtete ein 4,91-fach geringeres Risiko für Suizid und Suizidversuch bei Patienten mit rezidivierenden schweren Stimmungsstörungen, nicht nur bei bipolaren Störungen [34]. Dieses Ergebnis deutet darauf hin, dass die antisuizidalen Wirkungen der Lithiumbehandlung möglicherweise nicht nur mit den antidepressiven oder stimmungsstabilisierenden Wirkungen zusammenhängen, sondern auch mit einer Verringerung von Impulsivität und Aggression [35].

Daten aus insgesamt 13 randomisierten, kontrollierten Studien (RCTs, die nicht primär darauf ausgerichtet waren, eine Reduktion des Suizidrisikos zu belegen) zu Lithium mit 3836 Probanden, die Lithium (*n* = 1498) im Vergleich zu Placebo, einem stimmungsstabilisierenden Antikonvulsivum (heutige Bezeichnung: anfallssuppressives Medikament [ASM]) oder einem modernen Antipsychotikum (*n* = 2338 Teilnehmer) erhielten, wurden im Hinblick auf Suizidversuche und Suizide in einer Metaanalyse untersucht. Die gepoolte Odds Ratio (OR) betrug 0,49 zugunsten von Lithium (Z-Score = 2,46, *p* = 0,01; [14]). In absoluten Zahlen ausgedrückt kam es in einer weiteren Analyse dieser RCT-Daten unter der Behandlung mit Lithium bei 974 Patienten zu 3 Suiziden (0,31 % [95 %-Konfidenzintervall [KI] 0,06–0,90]), verglichen mit 21 Suiziden bei 1070 Patienten, die mit Placebo oder anderen Wirkstoffen behandelt wurden (1,96 % [KI 1,22–2,98], *p* = 0,01; [10]).

Zusammengefasst kamen mehrere systematische Übersichten und Metaanalysen insgesamt zu dem Schluss, dass eine Lithiumlangzeittherapie die Raten von Suizid und Suizidversuchen bei Patienten mit bipolarer Störung und schwerer depressiver Störung reduziert und das Risiko lebensbedrohlicher Suizidversuche und des Todes durch Suizid um 60–80 % verringert [14, 36]. In einem Metareview untersuchten die Autoren Smith und Cipriani [37] Daten aus 16 systematischen Reviews aus den Jahren 1980 bis 2017: Die Ergebnisse unterstreichen die suizidprotektiven Eigenschaften von Lithium in der Langzeittherapie.

## Lithium im Trinkwasser: Senkung des Suizidrisikos?

Es gibt immer mehr Hinweise darauf, dass selbst Spuren von Lithium, wie sie beispielsweise im Trinkwasser vorkommen, das Risiko für Suizid und Demenz bei lebenslanger Exposition senken können [38]. Replizierte Berichte aus verschiedenen Kontinenten beschrieben ein verringertes Suizidrisiko in geografischen Gebieten mit einer höheren Lithiumkonzentration im Trinkwasser. Bemerkenswert ist, dass solche Lithiumkonzentrationen eine tägliche Aufnahme erfordern, die weit unter der für die Behandlung bipolarer Störungen erforderlichen Menge liegt. Eine aktuelle systematische Übersicht von 15 Studien aus verschiedenen Ländern kam zu dem Schluss, dass tatsächlich eine inverse Beziehung zwischen der Lithiumkonzentration und der Suizidsterblichkeit besteht [39].

## Spezifische placebokontrollierte Studien

Bis heute gibt es nur zwei randomisierte placebokontrollierte Studien (RCT), die eine Reduktion von Suizidalität oder des Suizidrisikos als primären Outcomeparameter untersuchten. Die erste RCT-Studie wurde speziell konzipiert, um den Einfluss von Lithium auf suizidales Verhalten bei bipolaren und unipolaren depressiven Patienten (die kürzlich einen Suizidversuch überlebt hatten) über einen Zeitraum von einem Jahr zu untersuchen [40]. Alle Teilnehmer dieser deutschen Multicenterstudie im Rahmen des „Kompetenznetz Depression, Suizidalität“ erhielten eine Standardbehandlung, eine Gruppe zusätzlich Lithium, die andere Placebo. Die Analyse des primären Endpunkts ergab keinen signifikanten Unterschied in den Suizidhandlungen zwischen den mit Lithium und den mit Placebo behandelten Personen. Eine Post-hoc-Analyse ergab jedoch, dass alle vollendeten Suizide in der Placebogruppe auftraten, was einen signifikanten Unterschied in den Inzidenzraten darstellte (*p* = 0,049): Innerhalb eines Behandlungszeitraums von einem Jahr kam es in der Lithiumgruppe (*n* = 84) zu keinem Suizid, während in der Placebogruppe (*n* = 83) drei Suizide auftraten [40].

Eine weitere, neuere, placebokontrollierte RCT-Studie über 12 Monate wurde vorzeitig abgebrochen, da die Suizidereignisse bei 519 Probanden mit bipolaren oder depressiven Störungen (die kürzlich einen Selbstmordversuch überlebt hatten) aus einer Population der US-Veteranenadministration in den mit Lithium bzw. Placebo behandelten Fällen ähnlich waren [41]. Es wurde kein Gesamtunterschied bei wiederholten suizidbezogenen Ereignissen zwischen den Behandlungen festgestellt (Hazard Ratio 1,10). Eine Post-hoc-Analyse zeigte jedoch, dass das geschätzte 12-monatige Suizidrisiko 18,8 % für Lithium und 24,3 % für Placebo, das Risikoverhältnis (Risk Ratio) 0,78 und eine Risikodifferenz von −5,5 Prozentpunkten betrug [42]. Zu den methodischen Bedenken, die das negative Ergebnis im primären Outcomeparameter erklären könnte, gehörten niedrigere Serumlithiumspiegel als die in der klinischen Praxis üblichen (die Hälfte der Probanden hatte einen Lithiumspiegel von weniger als 0,5 mmol/l), die Zulassung zusätzlicher unkontrollierter Behandlungen und eine kurze Lithiumbehandlungsdauer von 12 Monaten [43].

## Besitzt Lithium Wirkungen zur Senkung akuter Suizidalität?

In einer aktuellen placebokontrollierten Studie wurde untersucht, ob eine Add-on-Lithiumtherapie (Zielserumspiegel 0,6–0,8 mmol/l) zur Standardtherapie („treatment as usual“, TAU) über einen Zeitraum von 5 Wochen bei hospitalisierten Patienten mit mittelschwerer bis schwerer Depression (unipolar oder bipolar) *und* Suizidgedanken oder suizidales Verhalten eine stärkere Verringerung der Suizidgedanken bewirkt als Placebo [44]. Lithium als Zusatztherapie zeigte im Vergleich zu Placebo keine überlegene kurzfristige Wirksamkeit bei der Reduktion von Suizidalität. Diese Ergebnisse deuten darauf hin, dass die Vorteile von Lithium bei Suizidalität möglicherweise auf Langzeitbehandlungen beschränkt sind [45].

## Fazit

In den vergangenen drei Jahrzehnten haben sich aus gänzlich unterschiedlichen Studienansätzen und Datenanalysen Hinweise verdichtet, dass eine Lithiumlangzeitbehandlung das Suizidrisiko und die Rate von Suizidversuchen bei Patienten mit bipolaren Störungen und vermutlich auch mit rezidivierenden unipolaren Depressionen senken kann. Die Betonung liegt hierbei auf „Langzeit“, denn die Befunde aus den zugrunde liegenden longitudinalen und epidemiologischen Untersuchungen stammen fast durchweg aus Studien, in denen die Patienten über mehrere Jahre hinweg mit Lithium behandelt wurden. Im Gegensatz zu dieser auch als Rezidivprophylaxe bezeichneten Therapie gibt es aktuell keine Befunde, die eine *akute* Senkung von Suizidgedanken und -handlungen nahelegen. Was für die rezidivprophylaktischen Lithiumeffekte im Hinblick auf die Dauer bis zum Wirkeintritt angenommen wird – nämlich, dass Lithium „Zeit braucht, um zu wirken“ (gewöhnlich mindestens 6 bis eher 12 Monate) – scheint nach aktueller Datenlage auch für die antisuizidalen Wirkungen von Lithium zu gelten. Nach derzeitiger Studienlage kann die Einnahme von Lithium zur Verringerung *akuter* Suizidalität nicht empfohlen werden. In der klinischen Praxis ist es daher wichtig, zwischen den längerfristigen und akuten, kurzfristigen Wirkungen von Lithium zu unterscheiden.

**Abb. 1:**
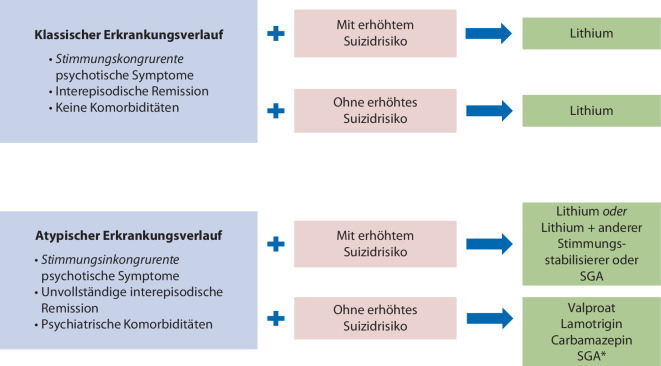
Algorithmus, wann eine Lithiumtherapie als medikamentöse Suizidprävention bei Patienten mit bipolaren und rezidivierenden depressiven Störungen in Betracht gezogen werden sollte. SGA* Second Generation Antipsychotics

Fehlende Kenntnis dieser langfristigen antisuizidalen Wirkungen, Unsicherheit darüber, wie diese in die klinische Praxis umgesetzt werden können, sowie generelle Vorurteile gegen eine längerfristige Lithiumbehandlung führen dazu, dass diese wichtige Therapieoption zur Reduktion suizidalen Verhaltens viel zu selten eingesetzt wird (exakte epidemiologische Daten wie häufig dies tatsächlich geschieht, liegen nicht vor). Ein Algorithmus, der zeigt, wann eine Lithiumtherapie als medikamentöse Suizidprävention bei Patienten mit bipolaren und rezidivierenden depressiven Störungen in Betracht gezogen werden sollte, ist in Abb. 1 dargestellt. Der Algorithmus soll Kliniker:innen bei ihrer Entscheidungsfindung unterstützen und berücksichtigt dabei auch Befunde zu wichtigen Aspekten des Krankheitsverlaufs und der Psychopathologie.

Insbesondere in akademischen Zentren in Nordamerika und Europa ist entgegen dem oben geschilderten Rückgang in der klinischen Praxis zuletzt ein zunehmendes Interesse an der Substanz Lithium zu beobachten. Dies ist auf zwei Dinge zurückzuführen: neue neurowissenschaftliche Erkenntnisse vor allem über die neuroprotektiven Effekte von Lithium [46] und seine einzigartigen klinischen Wirkungen bei Patienten mit schweren affektiven Störungen [17, 47].
